# Improved interface packing and design opportunities revealed by CryoEM analysis of a designed protein nanocage

**DOI:** 10.1016/j.heliyon.2022.e12280

**Published:** 2022-12-14

**Authors:** Stephen McCarthy, Shane Gonen

**Affiliations:** Department of Molecular Biology and Biochemistry, University of California, Irvine, CA 92617, USA

**Keywords:** CryoEM, Computational protein design, Structure, Nanomaterial, Symmetry, Icosahedral

## Abstract

Symmetric protein assemblies play important roles in nature which makes them an attractive target for engineering. *De novo* symmetric protein complexes can be created through computational protein design to tailor their properties from first principles, and recently several protein nanocages have been created by bringing together protein components through hydrophobic interactions. Accurate experimental structures of newly-developed proteins are essential to validate their design, improve assembly stability, and tailor downstream applications. We describe the CryoEM structure of the nanocage I3-01, at an overall resolution of 3.5 Å. I3-01, comprising 60 aldolase subunits arranged with icosahedral symmetry, has resisted high-resolution characterization. Some key differences between the refined structure and the original design are identified, such as improved packing of hydrophobic sidechains, providing insight to the resistance of I3-01 to high-resolution averaging. Based on our analysis, we suggest factors important in the design and structural processing of new assemblies.

## 1 Introduction

Symmetric protein complexes are found widely in nature, carrying out diverse functions including enzymatic transformations (Kyrilis et al., 2021; Zhang et al., 2003), transportation of substrates (Wiryaman and Toor, 2021), and encapsulation (Lončar et al., 2020; Nichols et al., 2021; Sutter et al., 2008). The utility of such complexes has made them an attractive target for bioengineering and computational protein design. Existing or newly developed nanomaterials are used to enhance existing functionality (Tan et al., 2021b), or aid in biochemical efforts to combat disease (Murray et al., 2022). These approaches rely on structural knowledge of the individual subunits that comprise the complex; evaluating the accuracy of predicted structures is therefore essential for optimizing their assembly, enhancing their effectiveness in downstream applications, and improving future designs.

**Figure 1:**
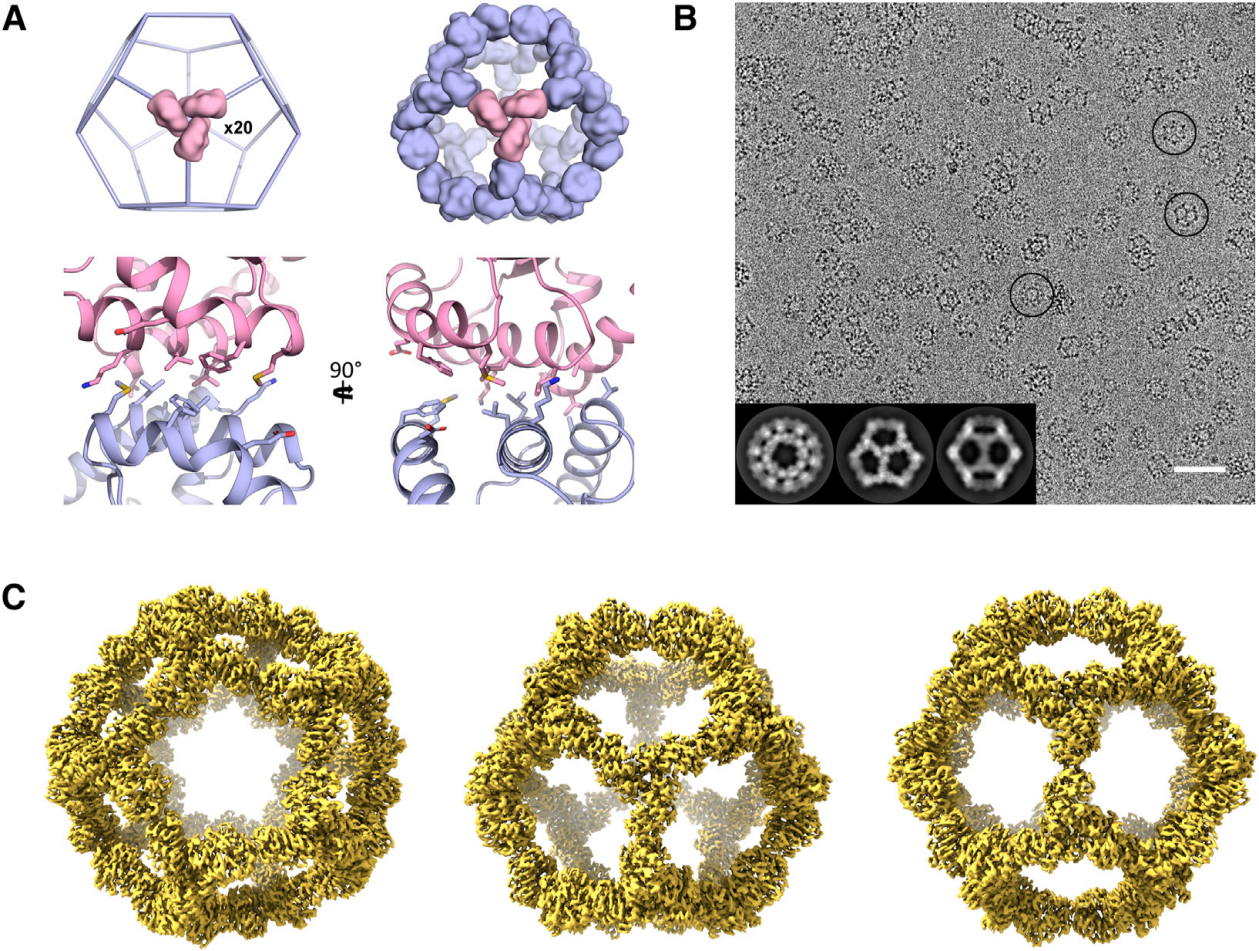
Overview of the I3-01 architecture and CryoEM. (A) (top) 20 trimeric aldolase molecules (aldolase in pink and identical copies in blue) were placed at the 3-fold vertices of icosahedral symmetry. (bottom) A primarily hydrophobic interface was designed between the subunits at the 2-fold axes to drive self-assembly. (B) CryoEM micrograph with frozen I3-01. Circled are examples of (from left to right) 5- 3- and 2- fold faces of the cage. (inset) 2D averages. Scale bar = 50 nm (C) 3.5 Å resolution map centered on the 5- 3- and 2- fold faces of the cage (left to right).

Here, we present the structure of a protein complex called I3-01, determined by CryoEM. This designed protein is derived from a trimeric 2-keto-3-deoxy-6-phosphogluconate (KDPG) aldolase (Fullerton et al., 2006), modified to introduce complementary hydrophobic interfaces resulting in self-assembly into a hollow cage with dodecameric geometry and icosahedral symmetry (Figure 1A) (Hsia et al., 2016). This protein has since been employed as a scaffold in vaccine design (Bruun et al., 2018; Guo et al., 2021; Liu et al., 2021; Tan et al., 2021a), as a vehicle for self-directed extracellular vesicle formation (Votteler et al., 2016), and in fluorescence microscopy for calibration (Akamatsu et al., 2020; Alamos et al., 2021; Dimou et al., 2019) or single-particle tracking (Xiang et al., 2021).

The I3-01 complex is large (26 nm diameter) and hollow, resulting in an assembly with a high solvent content, potentially making structural elucidation by X-ray crystallography challenging (Heras and Martin, 2005). Previous attempts to determine the structure of I3-01 by CryoEM have resulted in reconstructions that, while sufficient for backbone rigid body fitting to confirm the overall geometry of the design, have not been of high enough resolution to allow detailed comparison with the design model (Hsia et al., 2016; Shirasaki et al., 2022; Tan et al., 2021a; Votteler et al., 2016). Our high-resolution reconstruction allowed full flexible refinement of the design model against the EM map with side-chain placement and provides the first comparison of the design model to an experimental structure.

Here, we describe our structural data processing and analysis, and outline the differences and commonalities between the experimental atomic model to both the design and two X-ray crystal structures of the parent aldolase subunits. Our results suggest that although biochemically this protein complex is very stable (Hsia et al., 2016), small deviations likely coming from both the original aldolase enzyme and small interface motions combine to hinder high-resolution refinement through averaging.

The reconstructions and analysis described in this manuscript may aid in the design of new nanomaterials with novel functionality and in improvements to the stability of I3-01 and similar self-assembling designs.

## 2 Results

Low-resolution reconstructions of I3-01 (whereby alpha-helices can be discerned), can be calculated by averaging only a few thousand particles (Votteler et al., 2016) but a high-resolution reconstruction has remained elusive. We hypothesised that small conformational differences, combined with symmetry, may hinder high-resolution averaging. Using a newly-obtained dataset (Figure 1B), which allowed multiple rounds of 3D classifications, we determined a final map at an overall resolution of 3.5 Å calculated from nearly 150,000 particles (Figure 1C and Figure S1A and B). The final round of 3D classification also revealed a slightly more extended cage which was refined to 3.9 Å resolution (Figure S2). Using our 3.5 Å map, we performed fully flexible refinement of the model to obtain the final structure. We also calculated an additional reconstruction of the cage using the same particle set but without applied symmetry at an overall resolution of 4.2 Å (Figure S1C).

While the cage is biochemically stable (Hsia et al., 2016), the movements observed through our dataset, and other possible subtly-different movements compounded by symmetric averaging, likely hinder attempts at high-resolution reconstructions.

### 2.1 Structural comparison of the refined model to the design model

**Figure 2:**
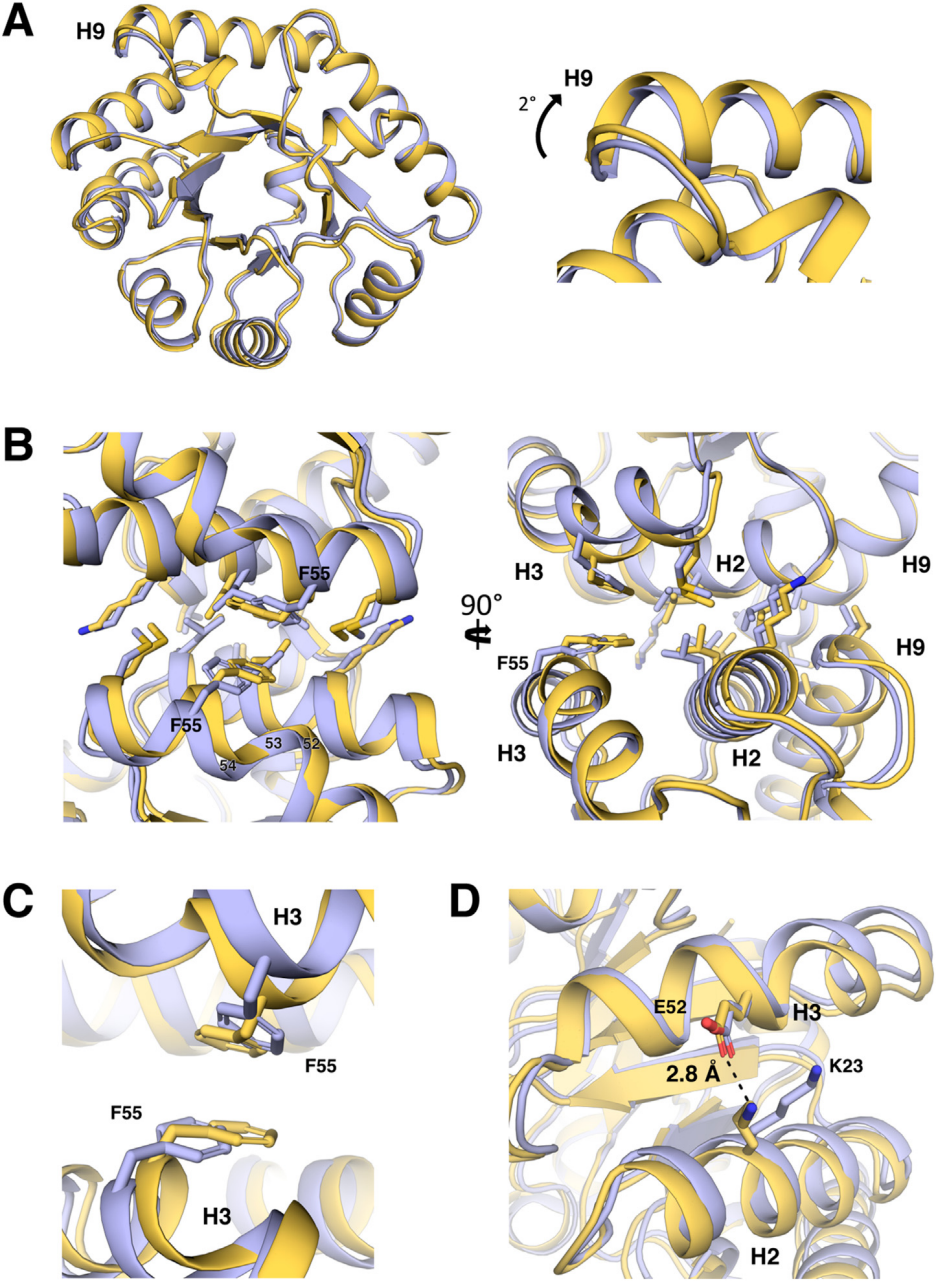
Structural comparison of experimental (gold) and design (blue) models. (A) Overlay of a single subunit of the experimental structure and the design model (left) and closeup of H9 highlighting the ≈2° rotation of the subunit (right). (B) Overview of the interface at the 2-fold symmetry axis. (C) Closeup on H3 highlighting the movement of F55. (D) Closeup of H2 and H3 showing the hydrogen bonding arrangement of K23 and E52.

The final reconstruction shows the expected geometry and particle size (diameter ≈26 nm) (Figure 1C). The experimental refined model, while close to the original design, shows some key backbone and sidechain movements that result in a tighter and more compact designed interface (Figure 2). These movements consist partly of inter-subunit rigid-body movements and partly of intra-subunit movements of the loops and helices, as indicated by the C_ɑ_ root mean-square deviations (RMSDs) for individual subunits before and after superposition (0.80 Å and 0.45 Å respectively). The rigid-body subunit movement consists principally of a small clockwise rotation (≈2°) about an axis approximately perpendicular to the 3-fold symmetry axis (Figure 2A), while the largest differences after superposition are found in the three helices by the designed interface on the 2-fold axis. These helices are defined by residues 18–32 (H2, C_ɑ_ RMSD = 0.58 Å), 53–60 (H3, C_ɑ_ RMSD = 0.59 Å) and 183–201 (H9, C_ɑ_ RMSD = 0.52 Å) (Figure 2B; helices and sheets are named as described in Figure S3B). By contrast, the β-strands that form the barrel-like core of the protein have considerably smaller displacements, with a typical C_ɑ_ RMSD in the range of 0.2–0.4 Å.

The combined effect of all these movements causes H2 to shift approximately 1 Å towards the N-terminal end of the helix and slightly down towards the C-terminal H9 (Figure 2B). H9 is similarly shifted down and slightly away from the interface by a similar amount. H3 moves slightly towards the N-terminal end of the helix and in towards the 2-fold symmetry axis. This movement is minimal in the N-terminal portion of the helix (typically 0.5–0.7 Å) but becomes much more pronounced after L53 (1.5–1.8 Å), which has the effect of accentuating the kink in the helix that is present at this position in the design model (Figure 2B). This movement causes a notable inward shift of the sidechain of F55 which serves both to increase burial of the hydrophobic aromatic ring and to form a more pronounced π-π stacking interaction with its symmetry mate across the 2-fold axis (Figure 2C).

The overall effect of these movements is to give the appearance of a slight clockwise rotation, as viewed along H2 through an axis perpendicular to the 2-fold symmetry axis (Figure 2B, right). The symmetry-paired subunit across the 2-fold symmetry axis correspondingly appears to move counter-clockwise. The movements of the helices towards the N-terminal end of H2, when set against the corresponding movement in the opposite direction of the subunit across the 2-fold symmetry axis, appear to slip past each other akin to a transform fault in plate tectonics.

Another difference is a movement of the sidechain of residue K23 on H2, which repositions such that it forms a hydrogen bond with the sidechain of E52 on H3 (Figure 2D). This interaction is also present in all six subunits of the parent aldolase crystal structure (Fullerton et al., 2006), but was not accounted for in the design model. Most other interface sidechains in the refined model retain a similar conformation to the design.

These movements also affect the trimeric interface centered on the 3-fold symmetry axis. The rigid-body rotational movement has the effect of slightly widening the interface on the outer edge of the particle and narrowing it on the inner face.

### 2.2 Comparative analysis of the models using Rosetta interface analyzer

In order to further understand the implications of the structural differences observed in the experimental model, we quantitatively analyzed the differences between the experimental and design model. Model refinement in Rosetta allows convenient use of Rosetta's InterfaceAnalyzer application (Stranges and Kuhlman, 2013), which calculates several metrics important in the creation and optimization of the original design, including binding energies, changes in solvent accessible surface area (ΔSASA), and shape complementarity (summarized in Table 1). Applying the InterfaceAnalyzer to the novel interface across the 2-fold symmetry axis shows that the movement of the interface helices increases the number of interface residues from 52 to 56, with a corresponding change in the calculated ΔΔG from roughly −30 to −37 Rosetta Energy Units (REU).

**Table 1:** Interface metrics as determined by the Rosetta InterfaceAnalyzer. ΔΔG is measured in Rosetta Energy Units (REU).

Parameter		Design model	Experimental model
ΔΔG (REU)	2-fold	−30.445	−36.724
	3-fold	−64.241	−76.519
No. of interface residues	2-fold	52	56
	3-fold	113	112
ΔSASA_total (Å^2^)	2-fold	1337.885	1312.648
	3-fold	2420.977	2484.479
ΔSASA_hydrophobic (Å^2^)	2-fold	1067.291	1097.332
	3-fold	1756.790	1730.666
Shape complementarity	2-fold	0.508	0.531
	3-fold	0.733	0.698

Despite the increase in the number of interface residues, the calculated total ΔSASA is decreased in the refined model, suggesting a more compact interface. The InterfaceAnalyzer provides a breakdown of the contribution to the overall ΔSASA from hydrophobic residues; this shows that while the total SASA is decreased compared with the design model, the calculated ΔSASA for hydrophobic residues is increased, suggesting that the refined model has improved burial of hydrophobic residues and provides a rationale for the observed differences between the design and refined models. The shape complementarity score is also increased in the refined model, further suggesting that the interface sidechain packing is improved in the refined model.

The small movements seen around the 3-fold symmetry axis are also reflected in changes in the InterfaceAnalyzer metrics. Unlike the designed interface, the calculated hydrophobic ΔSASA decreases despite an increase in the overall ΔSASA. While this metric, along with the shape complementarity score, is reduced, they are still within ranges observed for stable protein interfaces.

### 2.3 Comparison with the parent aldolase crystal structure

In order to further analyze the observed differences between the design and experimental models we compared both models to all available crystal structures of the parent aldolase. The basis for the design model was an X-ray crystal structure of KDPG aldolase from the hyperthermophilic bacterium *Thermotoga maritima* (PDB ID 1WA3) (Fullerton et al., 2006). The asymmetric unit contains six aldolase subunits as two trimers, and these subunits are very similar in structure but not identical. The design model backbone, which was created using a single chain of the trimer, is identical to Chain C of the crystal structure (C_ɑ_ RMSD = 0.00 Å) while the C_ɑ_ RMSD for the other chains ranges from 0.31–0.44 Å; the experimental model shows similar C_ɑ_ RMSD values (0.47–0.60 Å (Figure S3A).

A second X-ray crystal structure of this protein has been deposited in the PDB, although without an accompanying publication (PDB ID 1VLW) (JointCenter for Structural Genomics, 2004). This structure has three copies of the aldolase in the asymmetric unit, without forming a pseudo-symmetric trimer. When superposed on the different chains of the aldolase in crystal structure 1WA3, they show comparable C_ɑ_ RMSD values to the experimental model (0.51–0.66 Å), suggesting that the differences observed when comparing the experimental model to the design are within a range observed in different experiments, even on proteins with identical sequence. Since this second X-ray crystal structure does not have an accompanying publication, it is difficult to draw more detailed conclusions about the experimental conditions that may have led to any observed differences.

## 3 Discussion

The high-resolution structure of the designed, self-assembling protein cage I3-01 and the implications for design discussed below may aid in the successful design, structural characterization, and optimization of functional nanomaterials.

### 3.1 Implications for protein design

Using X-ray crystallography, the structures of many designed symmetric nanomaterials have been determined experimentally, and often show low RMSD to the design model (Bale et al., 2015, 2016). While the high overall similarity of the experimental model of I3-01 determined in this study to the design (C_ɑ_ RMSD <1 Å) demonstrates that accurate designs can be produced even for very large protein complexes, some features of the experimental model nonetheless suggest strategies that could further improve computational protein design. Our CryoEM-derived aldolase model can be added to the library of trimeric building blocks for future designs. Additionally, only one other designed cage has been determined to high-resolution to date by CryoEM (Liu et al., 2018, 2019), so the strategies presented here may prove useful for structural studies and design of protein complexes.

### 3.2 Selection criteria for designed interfaces

This study further supports the hypothesis that a smaller but well-packed interface is preferred to a larger interface with suboptimal packing. In the case of I3-01, this can be seen by the increase in shape complementarity score and number of interface residues in the experimental model of the designed interface, despite an overall decrease in the change in solvent accessible surface area on binding. The RosettaVIP (Void Identification and Packing) application may be an option for identifying interface regions that are underpacked and assist with design (Borgo and Havranek, 2012).

### 3.3 Allowing backbone movement during modelling

During the design of I3-01 the protein subunit backbone was treated as rigid, and consequently the movements of the helices observed in the experimental model would not have been modelled; allowing movement of the backbone during modelling could therefore potentially allow more accurate designs to be generated. Backbone movement is often restricted in modelling as it greatly increases the search space for design and is therefore computationally intensive, a problem that is especially challenging for large protein complexes with multiple interfaces. A compromise approach could be to separate the design process into discrete steps: treating the backbone as rigid in the first round of modelling followed by filtering of unpromising designs, then progressively allowing increasing backbone movement in subsequent rounds. While computationally expensive initially, for biochemically validated designs that resist high-resolution reconstruction or require additional stability, it may be beneficial to follow up with further design by allowing for small perturbations of the backbone near the interface to allow for tighter packing, to increase any potential π-π stacking arrangements or to make further mutations to increase hydrogen bonding networks.

The use of a shape complementarity score cutoff has been one of the main metrics in filtering out designs with suboptimal packing (Bale et al., 2016; King et al., 2014), and significant changes to shape complementarity following additional design steps can be filtered and looked at closely.

### 3.4 Explicitly accounting for π-π interactions in the Rosetta energy function

A notable feature of the experimental model of I3-01 was the formation of a π-π stacking interaction between the two interface F55 sidechains. While Rosetta's energy function REF15 accounts for electrostatic and van der Waals's interactions in determining sidechain energies, it does not model the permanent quadrupole on aromatic groups that contribute to π-π interactions, possibly leading to this interaction being overlooked during design (Alford et al., 2017). Explicit modelling of π-π and energies may therefore improve designs in which these interactions are a feature, and make clearer their contribution to the overall energy of the complex.

The π-π arrangement described for I3-01 above resembles a similar interaction from another successful self-assembling design – that of a six-fold symmetric protein designed to assemble into a two-dimensional protein array adhering to p6 symmetry (Gonen et al., 2015). In that study, it was shown that mutating only the interacting phenylalanine residues abolishes array formation. We predict that introducing π-π interactions could be a significant factor in designing similar successful assemblies.

## Declarations

### Author contribution statement

Stephen McCarthy: Conceived and designed the experiments; Performed the experiments; Analyzed and interpreted the data; Wrote the paper.

Shane Gonen: Conceived and designed the experiments; Performed the experiments; Analyzed and interpreted the data; Contributed reagents, materials, analysis tools or data; Wrote the paper.

### Funding statement

Shane Gonen was supported by National Institute of General Medical Sciences [R35-GM142797].

### Data availability statement

Data associated with this study has been deposited at PDB under the accession number ID 8ED3; EMD-28027; EMD-28028; EMD-28029.

### Declaration of interest’s statement

The authors declare no competing interests.

### Additional information

No additional information is available for this paper.

## Figures and Tables

**Table:** Key resources table

REAGENT or RESOURCE	SOURCE	IDENTIFIER
Deposited Data		
Structure of I3-01 refined into 3.5 Å map with I4 symmetry applied	This paper	PDB ID 8ED3
CryoEM map of I3-01 at 3.5 Å resolution with I4 symmetry applied	This paper	EMD-28027
CryoEM map of extended I3-01 with I4 symmetry	This paper	EMD-28028
CryoEM map of I3-01 with C1 symmetry applied	This paper	EMD-28029
Software and Algorithms		
Relion 3.1	Scheres, 2012a, 2012b	https://www3.mrc-lmb.cam.ac.uk/relion/index.php?title=Main_Page
MOTIONCOR2	Zheng et al. (2017)	https://emcore.ucsf.edu/ucsf-software
CTFFIND-4.1	Rohou and Grigorieff (2015)	https://grigoriefflab.umassmed.edu/ctffind4
Rosetta density tools	DiMaio et al. (2009)	https://www.rosettacommons.org/software
Coot	Emsley et al. (2010)	https://www2.mrc-lmb.cam.ac.uk/personal/pemsley/coot/
Q-scores	Pintilie et al. (2020)	https://github.com/gregdp/mapq
DAQ-scores	Terashi et al. (2022)	https://github.com/kiharalab/DAQ
MolProbity	Davis et al. (2007)	http://molprobity.biochem.duke.edu/
Rosetta IntefaceAnalyzer	Stranges and Kuhlman (2013)	https://www.rosettacommons.org/software
PyMol	Schrödinger, LLC	https://pymol.org/2/
UCSF Chimera	Pettersen et al. (2004)	https://www.cgl.ucsf.edu/chimera/
UCSF ChimeraX	Goddard et al. (2018); Pettersen et al. (2021)	https://www.cgl.ucsf.edu/chimerax/

## STAR methods

### Resource availability

#### Lead contact

Further information and requests for resources and protocols should be directed to and will be fulfilled by the lead contact, Shane Gonen (gonens@uci.edu).

#### Materials availability

This study did not generate new unique reagents.

#### Data and code availability

Structure data have been deposited in the Protein Data Bank and are publicly available as of the date of publication. Maps have been deposited in the Electron Microscopy Data Bank and are publicly available as of the date of publication. Accession numbers are listed in the key resources table. Original micrographs are available from the lead contact on request.

This paper does not report any original code.

Any additional information required to reanalyze the data reported in this paper is available from the lead contact upon request.

### Method details

#### CryoEM data collection

Purified I3-01 was frozen on Quantifoil grids using a Vitrobot Mark 3 (FEI) and screened for ice thickness and particle density. CryoEM data was collected using automated procedures using serial EM on a Titan Krios (Thermo Fisher) microscope. Super-resolution movies at a total dose of ∼40 e^−^ per movie (∼2.1 e^−^ per frame) were collected using a Gatan K2 Summit direct electron detector and used for all subsequent processing. Detailed data collection parameters are provided in Table S1.

#### CryoEM data processing

Micrograph stacks were imported into Relion (Scheres, 2012a; 2012b) and motion correction was applied using MOTIONCOR2 (Zheng et al., 2017) with two-fold binning to yield a pixel size of 1.31 Å/px. Contrast transfer function parameters were estimated with CTFFIND-4.1 (Rohou and Grigorieff, 2015). A total of 1,027 particles were picked manually from the 8,219 corrected micrographs and submitted for 2D class averaging for the purpose of creating templates for reference-based autopicking in Relion; six of the 2D class averages (representing a total of 407 particles) were selected. Using these class averages as references a total of 915,204 particles were picked.

The particles were divided into two subsets to facilitate better 2D class averaging. Two rounds of 2D classifications yielded 749,728 particles carried forward for 3D classifications. The initial model was created from six sets of 1,000 particles from 2D class averages representing the 2-fold, 3-fold, and 5-fold symmetric axes, and inverted to account for mirroring. C1 symmetry was applied during the reconstruction of the initial model, then I4 symmetry was applied in Relion. The initial model was low-pass filtered prior to 3D processing. Iterative rounds of 3D classification in C1 symmetry, followed by a single round of 3D classification in I4 symmetry without alignment, produced a final set of 147,349 particles which was refined with I4 symmetry applied. Successive rounds of CTF refinement and particle polishing, followed by post-processing, resulted in a final map of 3.47 Å resolution (Figure S1A).

One of the 3D classes in I4 symmetry without alignment was observed to be slightly ‘extended’ compared with the other classes; this class was separately refined and subjected to iterations of CTF refinement and post-processing to yield a map with an overall resolution of 3.87 Å resolution. The particles in this class were distributed throughout the dataset. This map was used to determine the ‘extended’ model conformation discussed in this work.

#### Model refinement

The design model was refined into the map using the fit-to-density tools in Rosetta (DiMaio et al., 2009). Symmetric fitting and scoring was enabled using the -symmetry:symmetry_definition flag and a symmetry definition file (generated by the make_symmdef_file.pl script provided with Rosetta) describing the icosahedral symmetry. The parameters for fitting were: -relax:fast -relax:jump_move true -edensity:mapreso 3.5 -edensity:grid_spacing 1 -edensity:sliding_window_wt 4 -edensity:sliding_window 3 -edensity:cryoem_scatterers -crystal_refine. A total of 100 refined models were generated, and the best scoring model was selected. An identical procedure was used with the ’extended’ map.

#### Processing in C1 symmetry

To ensure that enforcement of the icosahedral symmetry was not introducing distortions into the map, the final particle set was re-extracted, subjected to one round of particle polishing, then refined in C1 symmetry. The final resolution of this map, after post-processing, was 4.21 Å. The design model was fitted into this density with the same refinement protocol as for the icosahedral symmetry map, with the -symmetry:symmetry_definition flag omitted and the number of refined models set to 10. The refined C1 model did not deviate significantly from the model with applied icosahedral symmetry (Figure S1C).

### Quantification and statistical analysis

The fit to density was analysed visually in Coot (Emsley et al., 2010) and assessed with Q-scores (Pintilie et al., 2020) and DAQ-scores (Terashi et al., 2022). Model geometry was assessed using MolProbity, which also calculated Ramachandran statistics (Davis et al., 2007). Refinement and validation data are provided in Table S1. The InterfaceAnalyzer (Stranges and Kuhlman, 2013) in Rosetta was used for further analysis of the interfaces at the 2-fold and 3-fold symmetry axes (Table 1). RMSD values in the main text were calculated in PyMol (Schrödinger LLC, 2015). Figures were prepared in PyMol, UCSF Chimera (Pettersen et al., 2004), UCSF ChimeraX (Goddard et al., 2018; Pettersen et al., 2021), Microsoft PowerPoint (www.microsoft.com) and Adobe Photoshop (www.adobe.com).

## Appendix A Supplementary data

i301_structure_2022_supp_heliyon i301_structure_2022_supp_heliyon
