# Supplementary material for: Improved interface packing and design opportunities revealed by CryoEM analysis of a designed protein nanocage
Source: Heliyon. 2022 Dec 14;8(12):e12280. doi: 10.1016/j.heliyon.2022.e12280 (PMC9801105; doi:10.1016/j.heliyon.2022.e12280)
Supplement: i301_structure_2022_supp_heliyon [file mmc1.docx]

# Improved interface packing and design opportunities revealed by CryoEM analysis of a designed protein nanocage

Stephen McCarthy^1^ and Shane Gonen^1^*

^1^Department of Molecular Biology and Biochemistry, University of California Irvine, Irvine California 92617

*Correspondence to: Shane Gonen, University of California Irvine, McGaugh Hall, Irvine CA 92617. E-mail: [gonens@uci.edu](mailto:gonens@uci.edu)

Supplementary material:

**Figure S1.** CryoEM processing workflow of I3-01 using Relion and reconstructions in I and C1 symmetry

**Figure S2.** Extended map of I3-01 from a 3D classification subset

**Figure S3.** Comparison with parent aldolase structures, and isolated secondary structure elements

**Table S1.** Data collection, refinement and validation statistics for I3-01


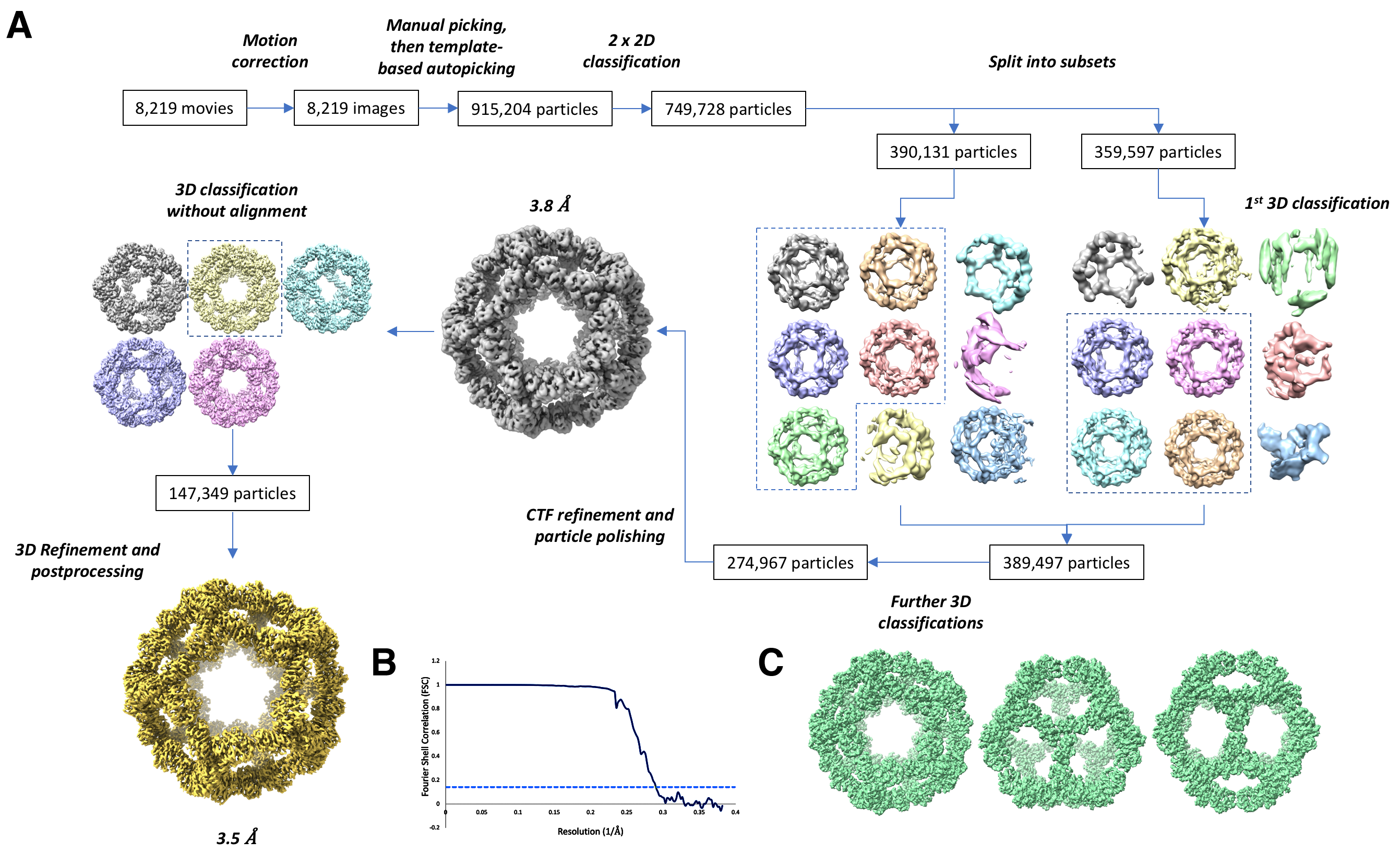


**Figure S1. CryoEM processing workflow of I3-01 using Relion. (A)** Particles were extracted from motion-corrected micrographs, split into subsets and put through multiple rounds of 2D and 3D classifications both with and without applied Icosahedral symmetry. An initial 3D model was calculated using the stochastic gradient descent algorithm in Relion using a small subset of 2D classified particles. The final 3.5 Å reconstruction was obtained after a round of 3D classification without alignment. **(B)** FSC curve used to determine the resolution of the map, with 0.143 used as the cutoff (dashed line). **(C)** Views of the map reconstructed from the final particle set without applied icosahedral symmetry.


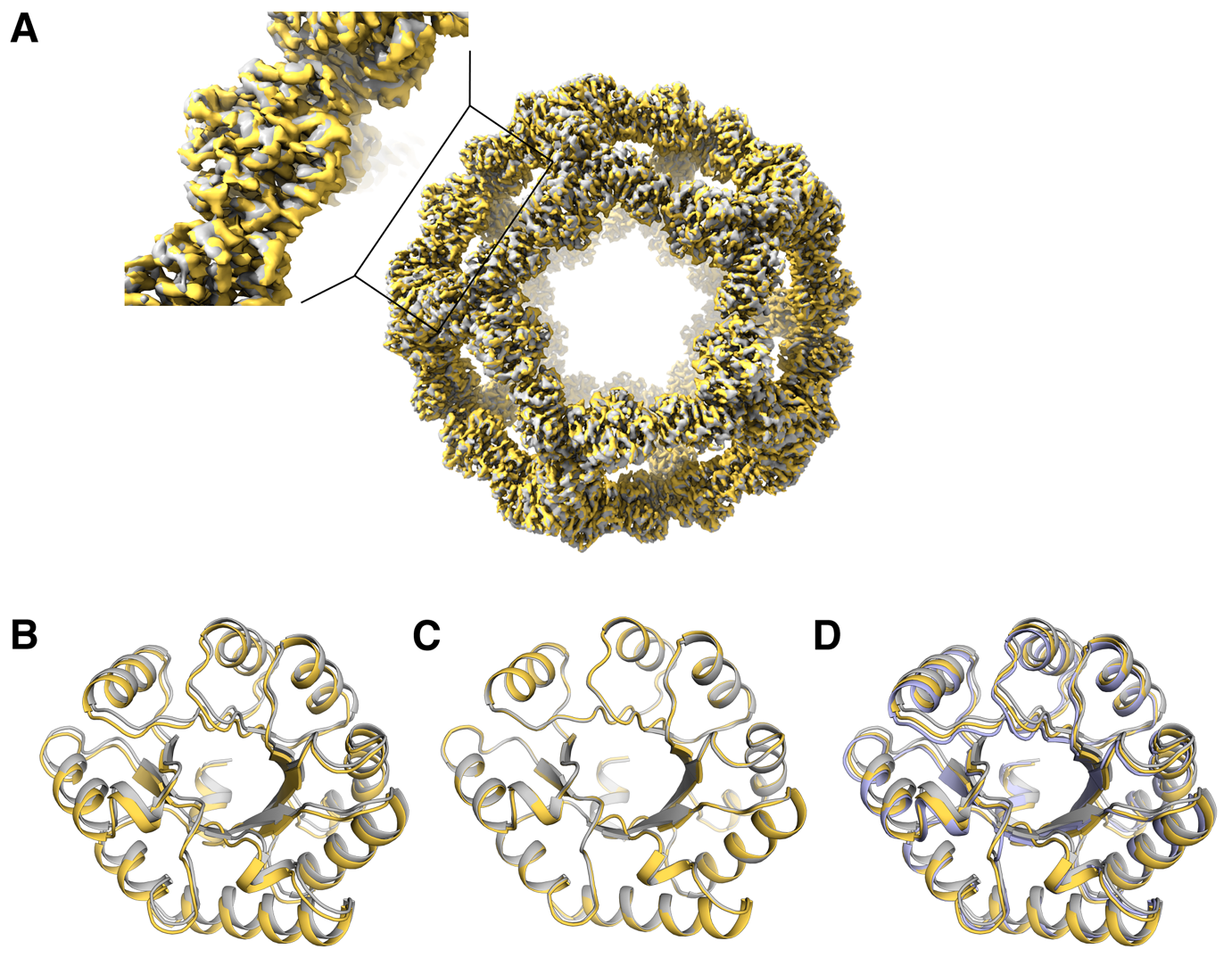


**Figure S2. Extended map of I3-01 from a 3D classification subset.** Particles from a 3D classification refined to 3.9 Å resolution reveal a slightly extended cage conformation. **(A)** Overlay of extended and the 3.5 Å resolution reconstruction (Extended map in silver) (inset closeup of one subunit). **(B)** Cartoon representation of a single subunit refined into both extended and high-resolution maps showing slight outward movement of the extended cage. **(C)** Superposition of the extended and 3.5 Å resolution model subunits showing minimal backbone movements **(D)** Overlay of the extended model, the 3.5 Å resolution model and the original Rosetta design of I3-01 (blue) showing repositioning of the extended model compared with the original design.


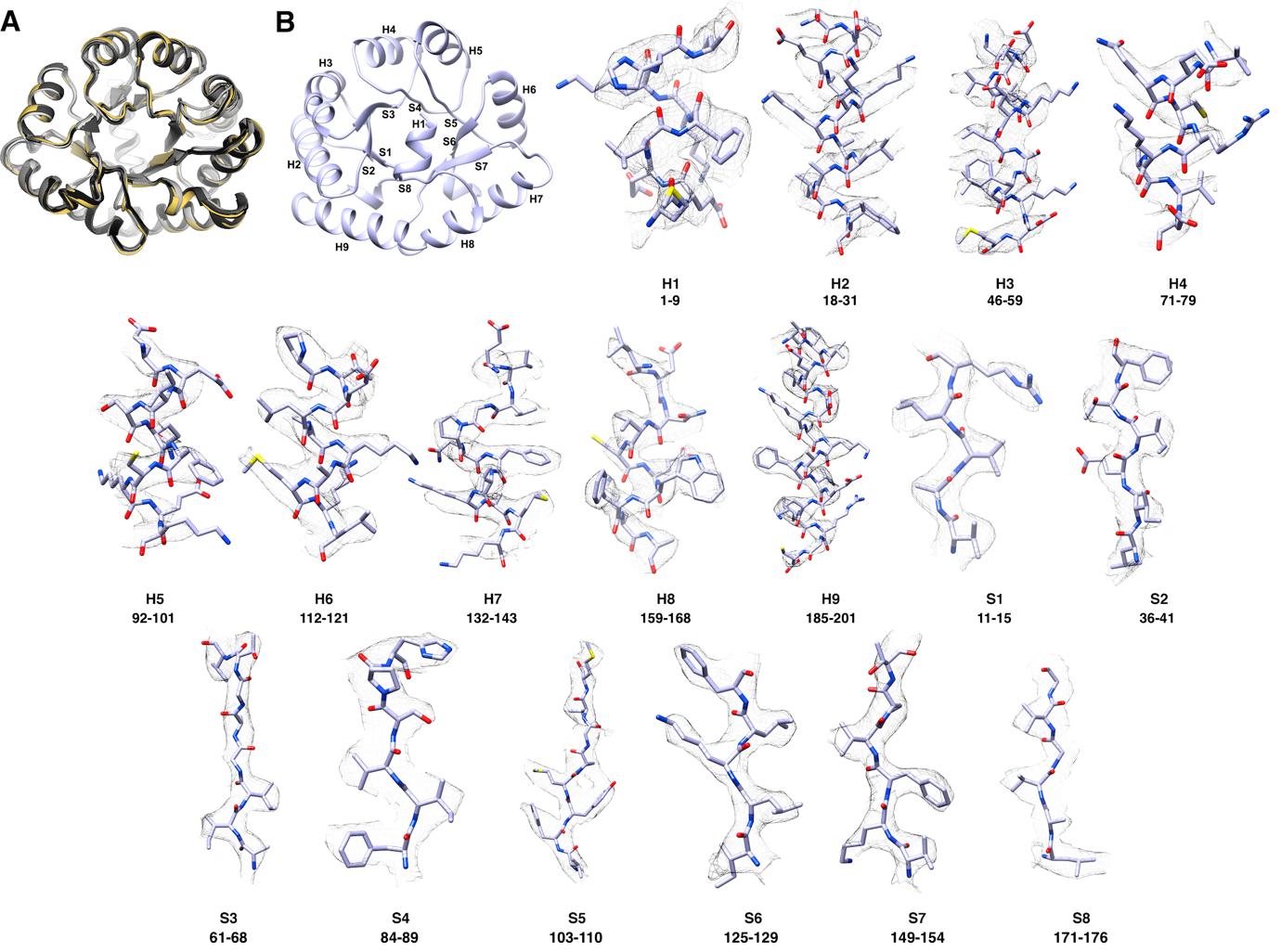


**Figure S3. Comparison with parent aldolase structures, and isolated secondary structure elements. (A)** Overlay of all sequence identical aldolases from the PDB (grayscale) and the experimental model (gold) **(B)** Secondary structure elements from a single subunit of the refined model labeled and fit to the experimental map. Helices and sheets are named according to the scheme at the top left.

**Table S1. Data collection, refinement and validation statistics for I3-01.** Maps and fitted models have been deposited in the Electron Microscopy Data Bank and Protein Data Bank, respectively, with accession codes EMDB-28027, EMDB-28028, EMDB-28029, and PDB ID 8ED3.

|  | **I3-01**  PDB 8ED3  EMDB-28027 |
| --- | --- |
| **Data collection and processing** | |
| Voltage (kV) | 300 |
| Electron dose (e^-^/Å^2^) | 2.1 |
| Defocus range (μm) | 1-3 |
| Pixel size (Å) | 1.31 |
| Symmetry | I |
| No. of micrographs | 8,219 |
| No. of initial particles | 915,204 |
| No. of final particles | 147,349 |
| Map resolution (Å) | 3.47 |
| FSC threshold | 0.143 |
| **Refinement and validation** | |
| Map sharpening *B* factor (Å^2^) | -161.242 |
| Model composition: | |
| Non-hydrogen atoms | 91,140 |
| Protein residues | 12,060 |
| Chains | 60 |
| Ramachandran plot: | |
| Favored (%) | 97.5 |
| Allowed (%) | 2.5 |
| Disallowed (%) | 0 |
| MolProbity score | 0.61 |
| Clashscore | 0.01 |
| Favored rotamers (%) | 97.5 |
| Poor rotamers (%) | 0 |
| Mean per-residue DAQ-score | 0.93 |
| Mean per-residue Q-score (expected at 3.5 Å) | 0.582 (0.502) |
